# FDG PET/CT for prognostic stratification of patients with metastatic breast cancer treated with first line systemic therapy: Comparison of EORTC criteria and PERCIST

**DOI:** 10.1371/journal.pone.0199529

**Published:** 2018-07-16

**Authors:** Edouard Depardon, Salim Kanoun, Olivier Humbert, Aurélie Bertaut, Jean-Marc Riedinger, Ilan Tal, Jean-Marc Vrigneaud, Maud Lasserre, Michel Toubeau, Alina Berriolo-Riedinger, Inna Dygai-Cochet, Pierre Fumoleau, François Brunotte, Alexandre Cochet

**Affiliations:** 1 Department of Nuclear Medicine, Centre Georges-François Leclerc, Dijon, France; 2 Le2i UMR 6306, CNRS, Arts et Métiers, Université Bourgogne Franche-Comté, Dijon, France; 3 Department of Biostatistics and methodology, Centre Georges-François Leclerc, Dijon, France; 4 BethIsrael Deaconess Medical Center, Boston Mass, United States of America; 5 Department of Medical Oncology, Centre Georges-François Leclerc, Dijon, France; Kyungpook National University School of Medicine, REPUBLIC OF KOREA

## Abstract

**Aim:**

Evaluate response and predict prognosis of patients with newly diagnosed metastatic breast cancer treated with first line systemic therapy using European Organization for Research and Treatment of Cancer (EORTC) criteria and PET Response Criteria in solid Tumours (PERCIST).

**Methods:**

From December 2006 to August 2013, 57 women with newly diagnosed metastatic breast cancer were retrospectively evaluated. FDG-PET/CT was performed within one month before treatment and repeated after at least 3 cycles of treatment. Metabolic response evaluation was evaluated by two readers according to both EORTC criteria and PERCIST, classifying the patients into 4 response groups: complete metabolic response (CMR), partial metabolic response (PMR), stable metabolic disease (SMD), and progressive metabolic disease (PMD).

**Results:**

With EORTC criteria, 22 patients had CMR, 17 PMR, 6 SMD and 12 PMD. With PERCIST, 20 patients had CMR, 15 PMR, 10 SMD and 12 PMD. There was agreement between EORTC and PERCIST in 84% of the patients. By log-rank analysis, metabolic response evaluated with both EORTC criteria and PERCIST was able to predict overall survival (p = 0.028 and 0.002 respectively). CMR patient group had longer median OS than patients in the combined PMR+SMD+PMD group (60 vs 26 months both with EORTC and PERCIST; p = 0.009 and 0.006 respectively). By multivariate analysis, CMR either with EORTC or PERCIST remained an independent predictor of survival.

**Conclusion:**

Metabolic response evaluation with EORTC criteria and PERCIST gave similar prognostic stratification for metastatic breast cancer treated with a first line of systemic therapy.

## Introduction

Metastatic breast cancer is an incurable disease in 25% of breast cancer patients, which makes it a major therapeutic challenge. Therefore non-curative treatment is used for prolonging life and reducing symptoms in order to improve quality of life [1]. For that purpose proper assessment of treatment response and prognostic stratification is essential in order to propose optimal and personalized therapeutic strategies [2].

Metabolic changes assessed with ^18^F-Fluorodeoxyglucose (FDG) Positron Emission Tomography/Computerized Tomography (PET/CT) has gained increasing interest for monitoring response to therapy in breast cancer, in both neoadjuvant and metastatic settings [3]. Several studies have shown FDG-PET/CT effectiveness in assessing response to systemic therapy in metastatic breast cancer [2,4–7]. However, generalization of the use of FDG PET/CT in this indication requires standardization of the response quantification methodology [8,9].

Currently, two sets of criteria to quantify anticancer treatment response in solid tumors have been described: the criteria developed by the European Organization for Research and Treatment of Cancer (EORTC) [10] and PET Response Criteria in Solid Tumours (PERCIST) [11]. EORTC criteria and PERCIST have different approaches for evaluation of treatment response; thus it is necessary to characterize, in each specific situation, the potential differences in outcome generated by the 2 sets of criteria in order to elucidate whether the criteria can be used interchangeably or give rise to significantly different results.

In addition we assessed whether the outcome evaluated in terms of overall survival in patients with metastatic breast cancer receiving first line systemic therapy is predicted by FDG-PET/CT metabolic response established either by EORTC or PERCIST 1.0 criteria.

## Materials and methods

### Patients

A retrospective analysis was performed in consecutive breast cancer patients referred to our institution for FDG-PET/CT from December 2006 to August 2013. All patients had histologically proven breast cancer, and were referred for breast cancer initial staging or recurrence. Inclusion criteria of this study were: (a) at least one distant metastasis with significant uptake on initial FDG-PET/CT (superior to liver or over surrounding background); (b) initial FDG-PET/CT had to be performed within one month before starting the first line of metastatic systemic treatment; (c) a second follow-up FDG-PET/CT had to be performed, after at least 3 cycles of therapy (or 3 months of endocrine therapy) and no later than 9 months after beginning of treatment.

Among the patients referred to our institution during this period, 159 patients had at least 2 FDG PET/CT scan with metastases shown on the baseline FDG-PET/CT scan and only 57 fulfilled all the inclusion criteria. As this study is retrospective, treating physicians used routine PET/CT results but were not aware of EORTC or PERCIST criteria results to make therapeutic decisions.

Patients were split into four different groups for further statistical analysis depending on histological type (ductal, lobular, other), histological grade of primary tumour (1–2 or 3), stage at the initial diagnosis (I, II, III or IV) or phenotype (HER2 +; Triple negative: HER2-, Hormonal receptor -; Luminal: HER2-/HR+). Immunostaging was performed on an automated immunostainer (Ventana XT, Tucson). We examined: oestrogen receptors (ER) using prediluted rabbit monoclonal antibody SP1, progesterone receptor (PR) using prediluted rabbit monoclonal antibody 1E2 and HER2 expression using prediluted rabbit monoclonal antibody 4B5.

ER and PR status were considered positive if tumor showed more than 10% of positive cells. HER2 status was considered positive according to HerceptTest scoring system if score was 3+. The 2+ scores had fluorescent in situ hybridization (FISH) (ZytoLight, SPEC HER2/CEN17 Dual Color Prob Kit) according to ASCO/CAP criteria.

All patients granted permission to review medical records at the time of PET/CT imaging according to our institution’s investigational review board guidelines for informed consent. The study procedures were in accordance with the ethical standards of the committees with responsibility for human experimentation (CPP Est I, France), with the Helsinki Declaration of 1975, as revised in 2008.

This manuscript has been reviewed and approved by internal ethic committee of the Centre Georges François Leclerc (IRB 00010311).

### FDG-PET/CT acquisition and processing

Whole-body FDG PET/CT, performed at baseline and during treatment, was acquired sequentially using a dedicated PET/CT system (Gemini GXL from December 2006 to December 2010 and Gemini TF from December 2010 to August 2013; Philips Medical Systems, Eindhoven, The Netherlands). Every patient included had their two PET/CT examinations acquired on the same system, if patients were scanned on the two different scanners they were excluded. Patients were instructed to fast except for glucose-free oral hydration for at least six hours before the intravenous injection of 5 MBq/kg (Gemini GXL) or 3 MBq/kg (Gemini TF) of FDG. Blood glucose levels were measured before the injection of the tracer to ensure levels below 10 mmol/L. PET was acquired 60±10 minutes following FDG injection, from brain to mid-thigh, with the patient supine. Conventional PET reconstruction was performed, emission data were all corrected for dead time, random and scatter coincidences and attenuation[12] before reconstruction with the RAMLA iterative method. Attenuation correction was calculated using a low-dose non-diagnostic CT acquisition (140 kV and 40–120 mA).

### FDG-PET/CT interpretation

FDG-PET/CT findings were interpreted by two experienced nuclear medicine physician blinded to clinical information. Usual clinical reporting visualisation protocol was used; orthogonal CT, PET and fused PET/CT images were displayed simultaneously, alongside a rotating maximum-intensity projection using Beth-Israel PET-CT viewer plug-in (http://petctviewer.org) for ImageJ software from FIJI (http://www.fiji.sc). This free and open source software being the tool of choice as it can gather various imaging information at the same time and allows reliable measures for statistical analysis [13,14]. Beth-Israel PET-CT viewer enable to draw outlines to define adapted regions of interest (ROI) in order to measure metabolic parameters for every single metastatic site. The Standardized Uptake Value (SUV) was calculated as follows:

$$SUV=\frac{C(t)}{\frac{A}{BW}}$$

Where BW = body weight (g), C(t) = radioactivity concentration in volume of interest at time T (MBq/mL), and A = injected dose (MBq). The attenuation-corrected PET emission scan was expressed in Bq/ml; SUV normalized by lean body mass to give SUL was also recorded [15]. For the purposes of EORTC et PERCIST the following metrics are defined: SUVmax, SULmax, SUVpeak, SULpeak (3D peak VOI determined (when possible) using a sphere with a diameter of approximately 1.2cm to produce a 1.0 ml spherical ROI positioned such that the average value across all positions within the lesion is maximised [15] [11]. In addition metastatic sites (bone, lung, liver, brain, lymph nodes, others) were registered for every ROI.

### Response evaluation with EORTC criteria

EORTC recommends using the pre-treatment scan to define regions on high FDG uptake that represent viable tumor. The whole tumour uptake should also be recorded (as no specific recommendations were given we recorded Metabolic Tumor Volume (MTV) and Total Lesion Glycolysis (TLG)). EORTC also recommends to use the same ROI volumes on subsequent scans, positioned as close to original tumour as possible and to measure mean and maximal tumour ROI counts per pixel per second calibrated as MBq/L[10]. We chose up to 5 of the lesions with the highest FDG uptake and up to two lesions per organ and measured the same lesions on the subsequent follow-up scan. As EORTC gives no information about the right number of lesions to measure: the number of 5 lesions was chosen knowing CT and PET studies agreed on measuring 3 to 5 lesions [11,16], and that RECIST and one of PERCIST’s definitions state 5 lesions should be measured.

All 5 targets SUVmax measurements were summed on each scan, giving ΣSUVmax. A percentage change in baseline, post-treatment summed SUVmax was calculated[17].

The patients were then classified into 4 response groups defined in EORTC as detailed in **Table 1**.

**Table 1:** EORTC metabolic response group definitions.

EORCT Metabolic response	
**Progressive metabolic disease (PMD)**	**Increase of at least 25% in ΣSUVmax or a visible increase in extent of FDG uptake (>20% in longest dimension) or appearance of new metastatic lesion(s)**
**Stable metabolic disease (SMD)**	**Response between PMR and PMD**
**Partial metabolic response (PMR)**	**Reduction in ΣSUVmax of at least 25%**
**Complete metabolic response (CMR)**	**Complete resolution of FDG uptake within all lesions (making them indistinguishable from surrounding tissue)**

ΣSUVmax: Sum of target lesions SUVmax

EORTC = European Organization for Research and Treatment of Cancer

#### Example of metabolic response evaluation

**Fig 1:**
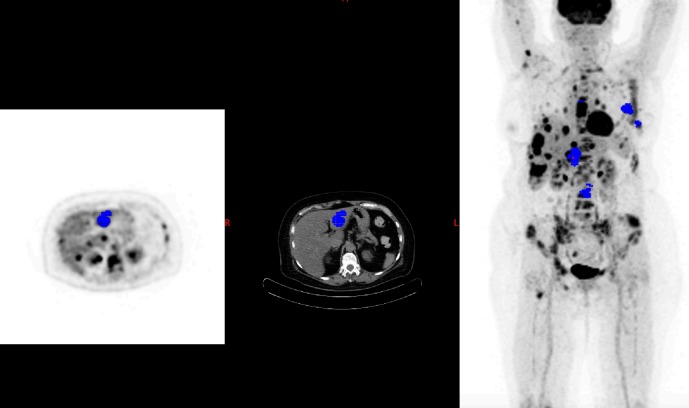
Baseline PET with 5 target lesions (EORTC). Blue spots corresponding to target lesions.

**Fig 2:**
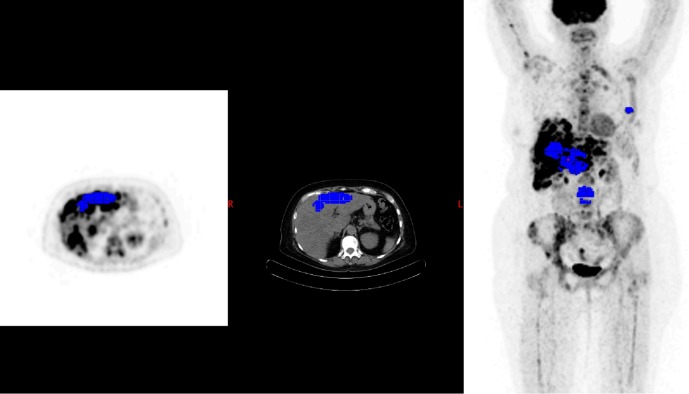
Follow up PET with new measures of the same 5 target lesions (EORTC). Blue spots corresponding to target lesions.

For one patient with EORCT criteria using Beth-Israel PET-CT viewer (**Fig 1** and **Fig 2** and **Table 2**):

**Table 2:** Summarised results of EORTC evaluation example.

	SUVmax baseline	SUVmax follow up
**Liver target 1**	**14.76**	**16.24**
**Liver target 2**	**16.41**	**16.31**
**Lymph node target 1**	**10.0**	**0 (disappeared)**
**Breast tumor target 1**	**10.75**	**10.09**
**Bone target 1**	**9.91**	**10.67**
**ΣSUVmax target lesions**	**61.83**	**53.31**
**ΔΣSUVmax target lesions**	**-13%**	

ΣSUVmax: Sum of SUVmax

ΔΣSUVmax: Variation (in percentage) of Sum of SUVmax between baseline and follow- up PET

In this case even if ΔΣSUVmax was between -25% and +25% the patient was considered as PMD (and not SMD) as new lesions appeared.

### Response evaluation with PERCIST

PERCIST recommends the use SUV normalization SUL rather than whole body mass, with no particular algorithm stated to calculate lean body mass. In our study we used Janmahasatian algorithm as suggested by Tahari *et al* [15,18]. The background area was drawn as a 3-cm-diameter spherical ROI in the right lobe of the liver as defined in the criteria. In patients with liver involvement, the background area was drawn in the descending thoracic aorta over 2 cm z-axis.

Measurable target lesion is the single tumour lesion having the highest SULpeak. “SULpeak corresponding at the SULmean in a 1.2cm diameter volume ROI positioned such that the average value across all positions within the lesion is maximised. Often this coincides with the location of SUV or SULmax [15]. The SULpeak has to be at least 1.5-fold greater than liver SULmean + 2 Standard deviations (SD). If SULpeak at baseline did not exceed the background value, the patient was not eligible for response evaluation with PERCIST.

Maximal SULpeak was recorded on each scan (typically located in the same lesion but could be located in a different lesion) and uptake modification was calculated as ΔSULpeak. Then ΔSULpeak was divided by baseline SULpeak x 100% to obtain ΔSULpeak percentage [11].

The patients were then classified into 4 response groups defined in PERCIST as detailed in **Table 3**.

**Table 3:** PERCIST metabolic response group definitions.

PERCIST 1.0 Metabolic response	
**Progressive metabolic disease (PMD)**	**Increase of at least 30% in SULpeak and an absolute increase of 0.8SULpeak units or a new FDG avid lesion**
**Stable metabolic disease (SMD)**	**Response between PMR and PMD**
**Partial metabolic response (PMR)**	**Reduction of at least 30% in SUL peak and an absolute drop of at least 0.8 in SUL peak units**
**Complete metabolic response (CMR)**	**Complete resolution of FDG uptake within all lesions to a level less than or equal to mean liver activity**

PERCIST = PET Response Criteria in solid Tumours

#### Example of metabolic response evaluation

**Fig 3:**
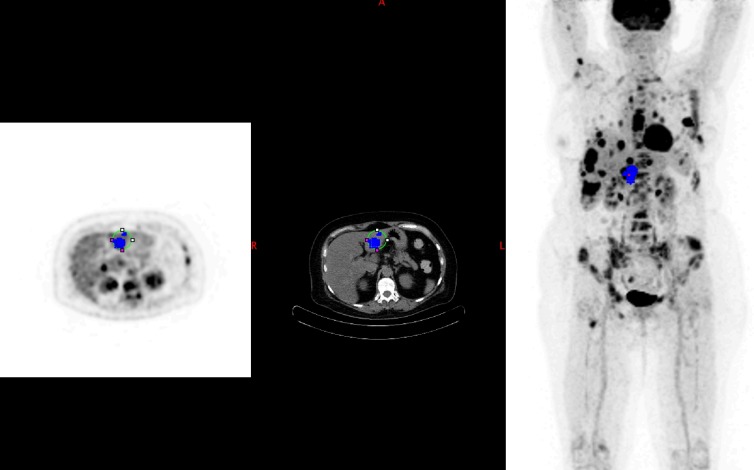
Baseline PET with a single target lesion (PERCIST). Blue spot corresponding to the lesion having the highest SULpeak (target lesion). As shown on this image the highest SULpeak target is located on the left lobe of the liver.

**Fig 4:**
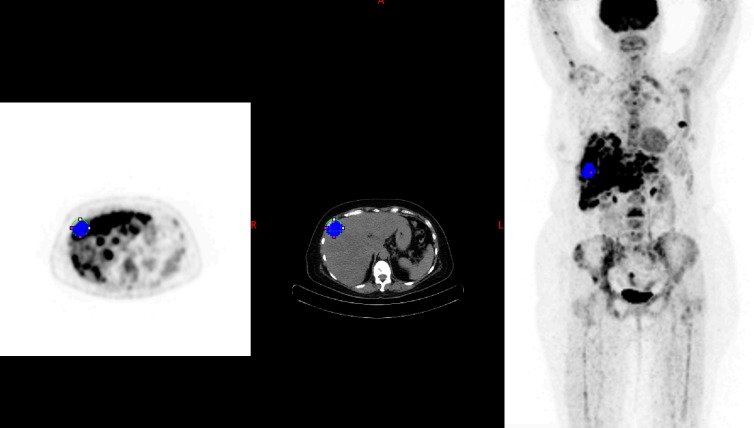
Follow up PET with a different location of the highest SULpeak target lesion (PERCIST). Blue spot corresponding to the lesion having the highest SULpeak (target lesion). This follow up scan having a different target lesion, now located on the right lobe of the liver.

For one patient with PERCIST criteria using Beth-Israel PET-CT viewer (**Fig 3** and **Fig 4** and **Table 4**):

**Table 4:** Summarised results of PERCIST evaluation example.

	SULpeak baseline	SULpeak follow up
**Target**	**8.1**	**8.31**
**ΔSULpeak target lesion**	**2.5%**	

ΔΣSULpeak: Variation (in percentage) of SULpeak between baseline and follow- up PET

Here as well (same patient as the EORTC example) even if ΔSULpeak was between -30% and +30% the patient was considered as PMD (and not SMD) as new lesions appeared.

### Statistical analysis

The simple and weighted kappa statistic (considering CMR>PMR>SMD>PMD) was used to measure agreement between readers and between response criteria. Reproducibility of both methods (EORTC and PERCIST separately) were evaluated by analysing simple and weighted kappa statistics between two trained readers blinded to clinical information.

Estimates of Overall survival (OS) were computed using the Kaplan-Meier method; a log-rank test was used to analyse the effect of metabolic response according to PERCIST and EORTC criteria on predicting OS, in the whole population, and in the subgroups according to phenotypes (triple negative, luminal, HER2+). Progression free survival (PFS) was not chosen to evaluate patient income as PFS is mainly determined by FDG-PET results. Cox regression multivariate analysis was performed to determine independent predictors of survival. Two multivariate models were tested, including metabolic response according to EORTC criteria or PERCIST.

## Results

Patient characteristics at the time of initial diagnosis of breast cancer are summarized in **Table 5**. The median age of the 57 patients was 60 years (range, 29–82 years). Average interval between baseline and follow-up FDG-PET/CT was 5 months (73 days to 268 days; σ = 47 days).

**Table 5:** Patient characteristics.

Characteristics	Number of patients (%) (Sample size = 57)
Histology	
Ductal	42 (73,7%)
Lobular	6 (10,5%)
Other	0 (0%)
Unknown	9 (15,8%)
Histological grade of the primary tumour	
1–2	28 (49,1%)
3	24 (42,1%)
Unknown	5 (8,8%)
Stage at the initial diagnosis	
I	7 (12,3%)
II	22 (38,6%)
III	10 (17,5%)
IV	18 (31,6%)
Phenotype	
HER2	9 (15,8%)
Triple negative	7 (12,3%)
Luminal	41 (71,9%)
Disease free interval	
0 (*de novo* MBC)	18 (31,6%)
<2 years	5 (8,8%)
>2 years	34 (59,6%)
Median No of metastatic disease sites (1^st^– 3^rd^ quartile)	7 (3–19)
Disease site	
CNS	0 (0%)
Lung	12 (21%)
Liver Bone	9 (15,8%)
	43 (75,4%)
Lymph nodes	38 (66,7%)
Other	5 (8,8%)

CNS = Central Nervous System; HER-2 = Human Epidermal Growth Factor Receptor-2; MBC = Metastatic breast Cancer

Between baseline and follow-up FDG PET/CT, 20 patients (35%) received anthracycline-based polychemotherapy (4 to 12 cycles, average: 7.3 cycles), sixteen (28%) received bevacizumab based therapy (4 to 11 cycles, average: 6.8 cycles), twelve (21%) received hormone therapy (3 to 8 months of hormone therapy, average 5 months) and nine (16%) received trastuzumab-based therapy (3 to 13 cycles, average: 6.8 cycles).

Agreements between EORTC Criteria and PERCIST for reader 1 and reader 2 are detailed in **Table 6** and **Table 7** respectively.

**Table 6:** Agreement between EORTC criteria and PERCIST (reader 1).

Response by	Response by PERCIST				
EORTC	CMR	PMR	SMD	PMD	Total EORTC
CMR	20	1	1	0	22
PMR	0	13	4	0	17
SMD	0	1	4	1	6
PMD	0	0	1	11	12
Total PERCIST	20	15	10	12	57

EORTC = European Organization for Research and Treatment of Cancer; PERCIST = PET Response Criteria in solid Tumours; CMR = Complete metabolic response; PMR = Partial metabolic response; SMD = Stable metabolic disease; PMD = Progressive metabolic disease

**Table 7:** Agreement between EORTC criteria and PERCIST (reader 2).

Response by	Response by PERCIST				
EORTC	CMR	PMR	SMD	PMD	Total EORTC
CMR	16	0	0	0	16
PMR	10	10	2	0	22
SMD	1	0	6	1	8
PMD	0	0	1	11	11
Total PERCIST	27	10	9	11	57

EORTC = European Organization for Research and Treatment of Cancer; PERCIST = PET Response Criteria in solid Tumours; CMR = Complete metabolic response; PMR = Partial metabolic response; SMD = Stable metabolic disease; PMD = Progressive metabolic disease

### Between readers

PERCIST had a higher agreements and corresponding kappa coefficients than EORTC between readers as shown in **Table 8.**

**Table 8:** Agreement and corresponding kappa coefficient between readers for EORCT and PERCIST.

Criteria	EORTC	PERCIST
Agreement reader 1–2	81%	86%
Simple Kappa	0.73	0.81
Weighted Kappa	0.82	0.86

EORTC = European Organization for Research and Treatment of Cancer; PERCIST = PET Response Criteria in solid Tumours

In summary there was disagreement in 19% (11 patients) between readers for EORTC:

- 5 patients having CMR with one reader and PMR with the other reader, explained by the fact that CMR definition by EORTC is based on visual interpretation of significant background. When there are still some very moderate uptake, one reader might consider it as not significant and other will consider it as just above surrounding background
- 1 patient having PMR with one reader and CMR with the other, this particular case explained by multiple very doubtful uptakes (diffuse brown fat uptake), it was hard to define if these uptakes were residual or not.
- 5 patients changing response category group from one reader to the other without being CMR for both readers.

There was disagreement in 14% (8 patients) between readers for PERCIST:

- 4 patients having PMR with one reader and CMR with the other reader, explained by difference of cut-off measurements (different ROI placement on liver depending on the reader): target lesion presenting a really low residual uptake will be measured as just above cut-off for one reader and just below for the other reader.
- 2 patients having SMD with one reader and CMR with the other one also explained by difference of cut-off measurements in addition to low baseline FDG uptake of the target lesion.
- 1 patient having PMR with one reader and CMR with the other reader for the same reasons as for EORCT disagreement on this particular case as there was many very doubtful uptakes.

### Between reporting methods

Agreement between EORTC and PERCIST for reader 1 and 2 were respectively 84% and 75% explaining our choice to continue further statistical analysis using only reader’s 1 data.

There was a disagreement in 16% (9 patients) between EORTC and PERCIST for reader 1 and the reasons for disagreement are outlined in **Table 9**. The number of patients with a change in the hottest lesion using PERCIST criteria was 16 (28%); the remaining 21 patients (57%) had the same hottest lesion.

**Table 9:** Reasons for disagreement between EORTC criteria and PERCIST (reader 1).

N° of patients	mR EORTC	mR PERCIST	Reason for disagreement
1	CMR	PMR	FDG uptake of the target lesion over PERCIST’s cut-off but visually not significant according to EORTC
1	CMR	SMD	Low baseline FDG uptake and lesion over PERCIST’s cut-off but visually not significant according to EORTC
4	PMR	SMD	Reduction in single-lesion SULpeak less than 30% and reduction in summed SUVmax over 25%,
1	SMD	PMR	Reduction in single-lesion SULpeak over 30% and reduction in summed SUVmax less than 25%,
1	SMD	PMD	Reduction in single-lesion SULpeak greater than reduction in summed SUVmax
1	PMD	SMD	Increase in single-lesion SULpeak less than 30% and increase in summed SUVmax over 25%,

EORTC = European Organization for Research and Treatment of Cancer; PERCIST = PET Response Criteria in solid Tumours; CMR = Complete metabolic response; PMR = Partial metabolic response; SMD = Stable metabolic disease; PMD = Progressive metabolic disease

### Results of PET/CT correlated with outcome measures

The median survival time was 30 months (range 7 to 71 months); 27 patients survived during follow up and 30 died.

The number of deaths during follow up depending on metabolic response using EORTC and PERCIST are outlined in **Table 10**.

**Table 10:** Number of deaths during follow up depending on metabolic response according to EORCT and PERCIST.

	EORTC	PERCIST
**CMR**	**8**	**7**
**PMR**	**12**	**10**
**SMD**	**2**	**4**
**PMD**	**8**	**9**

EORTC = European Organization for Research and Treatment of Cancer; PERCIST = PET Response Criteria in solid Tumours; CMR = Complete metabolic response; PMR = Partial metabolic response; SMD = Stable metabolic disease; PMD = Progressive metabolic disease

**Fig 5:**
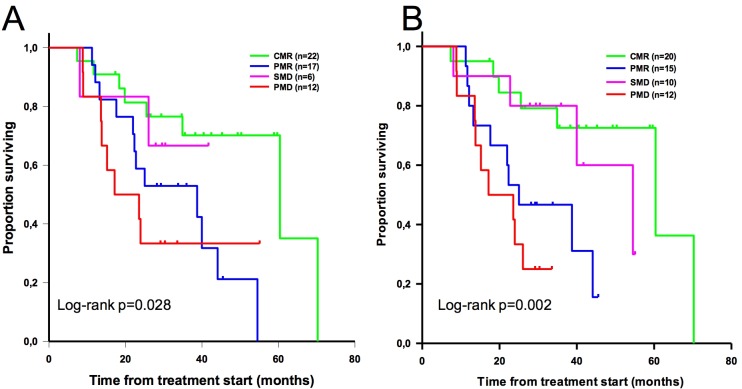
Overall survival stratified by metabolic response according to EORTC criteria (A) and PERCIST (B).

By log-rank analysis, stratification of metabolic response according to both EORTC criteria and PERCIST was able to predict survival (p = 0.028 and 0.002 respectively) (**Fig 5**). CMR group median OS in months with EORTC criteria was 60.4, 38.7 months in the PMR group, and 17.1 months in the PMD group (median OS in the SMD group was not computed because of the very limited number of patients in that group). The difference in median OS between the CMR and the PMR group, and between the CMR and the PMD group, were significant (p = 0,01 for both). With PERCIST, the median OS was 60.4 months in the CMR group, 25 months in the PMR group, 54.5 months in the SMD group, and 17.1 months in the PMD group. The difference in median OS between the CMR and the PMR group, and between the CMR and the PMD group, were significant (p = 0.009 and 0.001 respectively).

**Fig 6:**
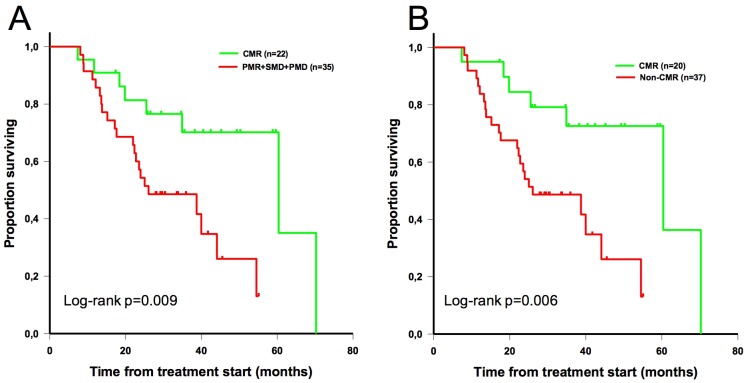
Overall survival stratified by metabolic response (complete or non-complete) according to EORTC criteria (A) and PERCIST (B).

Patients were also subcategorized between complete responders (CMR) and non-complete responders (non-CMR: PMR+SMD+PMD). Patients in the CMR group had significantly longer median OS than patients in the non-CMR group (60 vs 26 months; p = 0.009 with EORTC criteria; p = 0.006 with PERCIST) (**Fig 6**).

**Fig 7:**
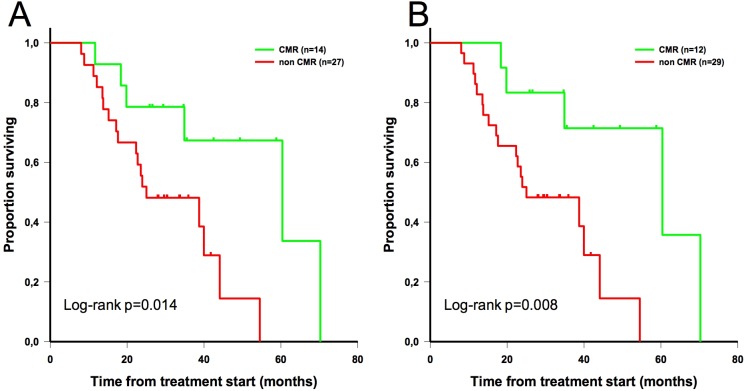
Overall survival stratified by metabolic response (complete or non-complete) according to EORTC criteria (A) and PERCIST (B) in patients with luminal breast cancer (phenotype with the best prognosis during at the time of the study).

Finally, patients were subcategorized by phenotype: luminal (HR+ HER2-, n = 41), HER2+ (n = 9), and triple negative (n = 7). In the luminal subgroup (phenotype with the best prognosis during at the time of the study), patients showing evidence of CMR had also significantly longer median OS than patients not showing evidence of CMR (60 vs 25 months; p = 0.014 with EORTC criteria; p = 0.008 with PERCIST) (**Fig 7**). Log-rank analysis was not significant in HER2+ and Triple Negative breast cancer (TNBC) small groups.

Results of the Cox proportional hazards regression model for prediction of death are reported in **Table 11**. By multivariate analysis, metabolic response stratified either with EORTC or PERCIST, and TNBC subtype remained independent predictors of death.

**Table 11:** Cox regression univariate and multivariate analysis for prediction of death (reader 1).

		Univariate analysis		Multivariate analysis (model 1)		Multivariate analysis (model 2)	
	n	OR [95% CI]	p	OR [95% CI]	p	OR [95% CI]	p
Age < median	28	1.31 [0.62–2.77]	0.47	1.35 [0.60–3.03]	0.46	1.52 [0.67–3.46]	0.30
Phenotype			0.08		**0.04**		0.12
HER2	9	1		1		1	
Luminal	41	6.72 [0.87–52.2]	0.06	6.22 [0.77–50.5]	0.08	5.34 [0.66–43.5]	0.11
Triple negative	7	11.88 [1.31–107.4]	**0.02**	14.78 [1.59–137.1]	**0.02**	10.2 [1.03–100.8]	**0.04**
Metabolic response (EORTC)			**0.04**		**0.03**		-
CMR	22	1		1			
PMR	17	3.2 [1.17–8.75]	**0.02**	3.51 [1.24–9.9]	**0.02**	-	-
SMD	12	1.56 [0.30–8.08]	0.59	1.32 [0.25–6.97]	0.74	-	-
PMD	6	4.13 [1.38–12.35]	**0.01**	4.46 [1.43–13.9]	**0.01**	-	-
Metabolic response (PERCIST)			**0.006**		-		**0.01**
CMR	20	1				1	
PMR	12	4.51 [1.45–14.0]	**0.008**	-	-	5.33 [1.64–17.4]	**0.004**
SMD	15	1.69 [0.44–6.48]	0.43	-	-	1.8 [0.45–7.11]	0.39
PMD	10	7.07 [2.13–23.4]	**0.001**	-	-	5.9 [1.7–20.5]	**0.004**
SBR III	24	1.08 [0.55–2.12]	0.83	-	-	-	-
*De novo* MBC	18	1.01 [0.46–2.23]	0.97	-	-	-	-

SBR III: Grade 3 tumors of Scarff-Bloom-Richardson grading system corresponding to a high grade breast cancer with aggressive potential

The results obtained for the small TNBC subgroup (N = 7; OR [95% CI]: 14.78 [1.59–137.1]), may be falsely significant and are probably over estimated due to the small sample size. However these results should be considered as a possible tendency and should be confirmed by larger prospective studies. [19]

## Discussion

Unlike for neoadjuvant therapy, measuring pathological response in the context of metastatic breast cancer is not possible. Thus, surrogate markers of tumour response to therapy and to survival are needed. Criteria based on tumour size and their modifications during therapy are limited because new-targeted therapies are more cytostatic than cytotoxic. Moreover, change in tumour size is not a good surrogate of bone lesions response [16]. For these reasons, evaluation of metabolic response of metastatic lesions to therapy using serial FDG PET/CT has gained increasing interest [3].

In the present study, we compared the performance to predict outcome of metabolic response evaluation methods with the 2 currently internationally recognized criteria (EORTC criteria and PERCIST; main characteristics of these criteria being summarised in **Table 12**) in patients with metastatic breast cancer receiving their first line of systemic therapy and evaluated if these methods were interchangeable.

**Table 12:** Summary of EORCT and PERCIST evaluation methods.

	EORTC	PERCIST
**Measurable parameters/lesions**		
**Measurable lesion (threshold definition)**	**Viable tumor (SUV> surrounding background)**	**SULpeak >1.5 liver SULmean + 2DS**
**Target lesions**	**Viable tumors; no number given (5 in study)**	**Highest SULpeak lesion (1 lesion)**
**Measured parameter for metabolic response evaluation**	**ΔΣSUVmax target lesions**	**ΔΣSULpeak target lesion**
**Response criteria**	** **	** **
**CMR**	**No viable tumor**	**No lesion above > threshold**
**PMR**	**Reduction of minimum of 25% in ΔΣSUVmax**	**Reduction of minimum of 30% in target measurable tumor 18F-FDG SUL peak**
**SMD**	**Not CMR, PMD or PMR**	**Not CMR, PMD or PMR**
**PMD**	**Increase in ΔΣSUVmax >25% or new lesion**	**Increase in SULpeak >30% or new lesion**

EORTC = European Organization for Research and Treatment of Cancer; PERCIST = PET Response Criteria in solid Tumours; CMR = Complete metabolic response; PMR = Partial metabolic response; SMD = Stable metabolic disease; PMD = Progressive metabolic disease

Our main results are that despite discrepancies, criteria show good agreement; although not interchangeable do provide comparable results in prognostic stratification, with complete response being a major predictor of survival in the whole population.

Comparison of PERCIST and EORTC criteria has been performed in metastatic colorectal and small cell lung cancer [17,20]. Skougaard’s study of metastatic colorectal cancer showed similar responses and similar response measures between EORTC and PERCIST OS outcomes and good agreement on best overall metabolic response (best overall metabolic response being the best metabolic response considered during a patient’s treatment course from consecutive scans; kappa coefficient = 0.76) and similar significant differences in median OS between response group[17]. Ziai’s study of small cell lung cancer showed perfect agreement between EORTC criteria and PERCIST[20]. In our study, EORTC criteria and PERCIST also showed good agreement. EORTC criteria and PERCIST disagreed on the response evaluation for 9 patients (16%). The differences in response evaluation are explained for CMR by metastases with low FDG uptake on follow-up scan, not significant according to EORTC criteria, but above the cut-off according to PERCIST (**Table 9**). CMR disagreement is the most concerning as complete response appears to be the most powerful predictor of survival in our study. Other discrepancies are less troublesome from a clinical perspective; they were more generally explained by the differences in quantification parameters (SUVmax versus SULpeak and multiple lesions versus single lesion) and in response cut-off (25% versus 30%).

Despite their discrepancies, EORTC and PERCIST gave comparable results in response evaluation and prognostic stratification. However, comparison of results between two readers show that PERCIST has higher agreement and kappa coefficients than EORTC suggesting a higher reproducibility of PERCIST between readers. The higher reproducibility of PERCIST is probably explained by the differences of definitions between the two methods:

- EORTC gives more freedom of choice than PERCIST for many measurable parameters:
  - No recommendations on the number of targets needed for response calculation. We choose precisely 5 in this study, which probably enhanced agreement between readers.
  - Either SUVmax or SUVmean can be measured for the target lesion. We choose SUVmax instead of SUVmean as it has been shown that maximum values are more resistant to partial volume effect than mean values [11] and have a lower inter-observer variability [21].
  - No precise definition of the minimum background SUV that viable tumours should exceed in order to be qualified as a target lesion.

Thus EORTC reliability is theoretically affected by decreased intraobserver reproducibility.

- PERCIST is giving more precise definitions, giving less freedom to the reader to choose and measure parameters:
  - The reader should consider the most metabolically active part of the single most FDG active tumour.
  - The reader has to use SULpeak and SULpeak only. This choice of using SULpeak is justified as normalizing SUV by the lean body mass avoids artificially high organ SUVs in obese patients as fatty tissues have a much lower FDG uptake than organ tissue. By using SUL instead of SUV, metabolism measures are more consistent between patients with different body types [22,23].
  - The reader uses SUV (SUL) peak, which is mathematically more robust than either SUVmax or SUVmean as SUVpeak is spatially averaging the voxel intensity over a fixed small volume rather than over a large not very well defined region as in SUVmax, which is subject to random noise.
  - Detailed background definition for target lesions: SULpeak has to be at least 1.5 times greater than the SULmean + 2SDs of a 3cm spherical ROI in normal right lobe of liver, which will also lower the test-retest variance.

Thus PERCIST detailed criteria definitions make it more robust than EORTC [11], explaining PERCIST’s higher agreement and kappa coefficient between readers in our study.

The cut-off values are also different between EORTC criteria and PERCIST (25% and 30% respectively), but this difference was a cause of discrepancies in only 5 patients in our study. These cut-off differences are explained by when these two studies were held and on the different types of pathologies they focused on.

Both methods defined their cut-off by reviewing studies aiming to establish correlation between alterations in FDG uptakes after chemotherapy and conventional response assessment. EORTC published in 1999 focused on 10 different studies that included Glioma, Medulloblastoma, Head and neck carcinoma, Breast Cancer and Colorectal liver metastases and the mean cut-off for these different studies was 25%. PERCIST published in 2009 showed by reviewing various studies that for Lymphoma, Lung cancer, Sarcoma, GIST, Gastric an Ovarian carcinoma (pathologies that were not included in EORTC) a decrease less than 30% in SUV was not enough to predict a better outcome. Therefore suggesting using a 30% cut-off.

Our daily practice suggests that PERCIST is easier to apply, as it requires only one measurement per FDG-PET/CT scan with unequivocal guidelines in all aspects of the evaluation procedure. The finding that evolution of only one lesion predict outcome; thus, clearly progressive disease in any one lesion is representative of a global progression of the disease is interesting and consistent with literature about heterogeneous response having similar outcome as progressive disease. For patients having clone metastases we could consider that the whole disease is progressing which informs us on the outcome and orientates us on treatment modifications. However for patients that may be presenting heterogeneous metastatic lesions PERCIST is still helpful predicting the outcome but is probably not giving enough information in order to modify treatment, as every metastasis will probably have different response to the treatment.

One of the major findings in our study is that the overall survival time was nearly 3 times as long in patients showing CMR on PET/CT as in patients showing any other response. Moreover EORTC and PERCIST identify CMR very similarly as CMR is a complete resolution of FDG uptake within all lesions for EORTC or to a level less than or equal to mean liver activity for PERCIST. Regarding CMR our results are comparable and appear consistent with previous studies. In clinical routine, presence or absence of progression is usually considered to adapt therapeutic strategies. However, our findings emphasize the importance of CMR rather than the absence of progression as the objective of the treatment.

Our study has some limitations. It is retrospective, with patients under different systemic therapies. It has been shown in the neoadjuvant setting that kinetic of metabolic response can be influenced by drug regimen [24]. Moreover, tumour response to neoadjuvant endocrine therapy seems to be slower than with neoadjuvant chemotherapy [25,26]. However, this therapeutic heterogeneity is less problematic in our study since we evaluated mid-response to treatment, rather than early response.

We considered patients with newly diagnosed metastatic disease, *de novo* or after therapy with curative intent. However, most of patients experiencing recurrence had a metastatic-free interval longer than 2 years, which has been shown to be a comparable situation in term of prognosis, with *de novo* metastatic disease [27].

## Conclusion

In patients with metastatic breast cancer, the effectiveness of first line systemic therapy can be evaluated by baseline and interim FDG-PET/CT scans using EORTC criteria and PERCIST although these methods are not interchangeable they do provide comparable results in prognostic stratification. Prognostic stratification, with a complete metabolic response being the main criteria to identify women with prolonged survival.

PERCIST criteria seem more straightforward and are more reproducible between readers than EORTC. However it needs to be confirmed by prospective studies in order to lead to a consensus on the best way to evaluate FDG PET/CT response with the aim of monitoring and adapting the treatment in metastatic breast cancer.
